# Charity, Capacity, Continuity

**Published:** 1896-06-13

**Authors:** 


The Hospital

An Institutional Journal of

The Medical Sciences and Hospital Administration, with

An Extra Nursing Supplement.

Vol. XX.—No. 507.] [June 13, 1896.

### Charity, Capacity, Continuity.

The note of "Charity in extremis" has often been played upon. "Charity in excelsis" is a much more inspiring note, and expresses in much fuller measure the true state of affairs. That the needy sick, all of them, in a nation of nearly forty millions of inhabitants, should be taken out of their poverty and necessity the moment their cases become urgent, and should, as if by the stroke of a magician's wand, be immediately placed in circumstances where the highest science, the truest tenderness of feeling, and every possible material resource that money can buy, are placed at their disposal, is a consummation which may with perfect sobriety be called one of the most striking of all the striking phenomena of the civilisation of the nineteenth century. This is what is actually done, with a number amounting to millions of sick persons, every year in Great Britain. Our hospital system copes with these numbers, copes with them in their deadly helplessness, copes with them without friction, without strain or worry to the sick themselves, and with a success which is truly magnificent in soothing, relief, and cure.

What reward has civilisation itself for all this labour, skill, and moral worth? For it is labour, skill, and moral worth which have built up this one great pillar of modern British life. What is the result, if any, of a profitable kind, to that portion of the community which provides the benefits for the sick but does not directly share in them? The reward in the first instance is that of seeing a great and quite indispensable work well done, ideally well done; and done with practically no waste of resources. This work, so done, is honourable to our practical capacity, and an undeniable evidence of the soundness of the moral heart of modern Britain. Let outsiders say what they will of us; let us say, and even cynically think, what we will of ourselves, we cannot be hopelessly corrupt or base, if of our own free will and without any compulsion whatsoever, we show our poor and sick brethren so truly great an object lesson every year in the art of doing as we would be done by. It is not a bad thing, fellow Britons; it is not a poor or a paltry or a mean thing which we do every year. It is a great thing and a noble; and it is right that we should see it in its true quality, and should mark out its proportions exactly as they are. Those who are not already quite familiar with its proportions and its quality will find the information and illumination they are in need of in the pages that follow.

But a greater reward than the approval of conscience results from our scientific and charitable work in hospitals. We get the material there in abundance, and we get the necessary material resources, which enable us to plumb disease in almost every form to its very depths; and not disease only, but health and life as well. By means of hospitals we have made out a full measure and an actuality of knowledge of ourselves as human beings such as no previous century has ever attained to. Here we stand to-day in the midst of a cultivated country of biological, pathological, and therapeutical knowledge which, for fulness, clearness, and depth, is a monument of the work of our century unsurpassed for its splendour and its value to mankind. This is our greater reward.

Will our civilisation live, or will it go down before irresistible forces as the civilisations of Greece, Rome, and Egypt have done long before it? When we ask ourselves this question, we scarcely know even where to begin to look for an answer. The work of the hospitals points with an answering finger. What do we find combined in that work? Science and high motive, knowledge and noble intention! What are the factors of continuity, of permanence in an individual man or woman? Are they not these two—knowledge and nobility of motive? If this nation, this civilisation, carries out its work in every other department of its activity with the same combination of knowledge and high motive it has exhibited in its hospital activities, we hold that its corruption will be impossible, its extinction will be impossible, its continuity and its permanence will be established through ages which need not be numbered.

The future Lecky who shall write the "History of English Morals" will most assuredly devote a great part of his work to the practical morals which founded, maintained, and carried out the daily work of hospitals, and to the reflex influence of that work upon the personal, social, and political character of the British peoples. Does any man seek to know what is the real inner character of the worthiest members of the British race at the present moment? Let him fill his mind and heart with the knowledge of all that is being done, for science, for humanity, and for the progress of the race in our manifold hospitals. He will then have some true idea of what plain John Bull at his best really is.

Never has this journal reported the hospitals as being *in extremis*. Always it has held that they must be kept, not only out of the depths but out even of the shallows—out of the water altogether, and placed, like a noble city set on a hill, where everybody may see and admire. When they are seen, when their work is seen, is known, is understood, one feels that it is an impertinence, an offence to "beg" for them. We have always been opposed to the "begging system." Make the hospitals and their work known; let those who want to know it read through our present number; let them visit repeatedly and study the work of the hospitals in the hospital wards. Then there can be no question that ample resources for all real needs will be placed in the hands of those who make themselves responsible for the helpless sick. Nothing less than a pillar of civilisation, one of the noblest and most enduring of all its pillars, is represented by the charities and work of hospitals.

